# Pain predictability reverses valence ratings of a relief-associated stimulus

**DOI:** 10.3389/fnsys.2013.00053

**Published:** 2013-09-24

**Authors:** Marta Andreatta, Andreas Mühlberger, Evelyn Glotzbach-Schoon, Paul Pauli

**Affiliations:** ^1^Department of Psychology (Biological Psychology, Clinical Psychology and Psychotherapy), University of WürzburgWürzburg, Germany; ^2^Department of Experimental Psychology (Clinical Psychology and Psychotherapy), University of RegensburgRegensburg, Germany

**Keywords:** backward conditioning, forward conditioning, implicit and explicit responses, pain relief, threat unpredictability

## Abstract

Relief from pain is positively valenced and entails reward-like properties. Notably, stimuli that became associated with pain relief elicit reward-like implicit responses too, but are explicitly evaluated by humans as aversive. Since the unpredictability of pain makes pain more aversive, this study examined the hypotheses that the predictability of pain also modulates the valence of relief-associated stimuli. In two studies, we presented one conditioned stimulus (_FORWARD_CS+) before a painful unconditioned stimulus (US), another stimulus (_BACKWARD_CS+) after the painful US, and a third stimulus (CS−) was never associated with the US. In Study 1, _FORWARD_CS+ predicted half of the USs while the other half was delivered unwarned and followed by _BACKWARD_CS+. In Study 2, all USs were predicted by _FORWARD_CS+ and followed by _BACKWARD_CS+. In Study 1 both _FORWARD_CS+ and _BACKWARD_CS+ were rated as negatively valenced and high arousing after conditioning, while _BACKWARD_CS+ in Study 2 acquired positive valence and low arousal. Startle amplitude was significantly attenuated to _BACKWARD_CS+ compared to _FORWARD_CS+ in Study 2, but did not differ among CSs in Study 1. In summary, predictability of aversive events reverses the explicit valence of a relief-associated stimulus.

## Introduction

Reliable predictions of painful or threatening events modulate the perception of such events. Namely, both humans and mice respond to an aversive auditory stimulus with greater amygdala activation when such stimulus was presented unpredictably than when it was predictable (Herry et al., 2007). Moreover, human participants rate a painful stimulus more intense and more negative when they cannot reliably predict its delivery by means of a visual cue (Carlsson et al., 2006). In the same vein, a context in which a painful electric shock was unpredictably delivered induced higher anxiety level and potentiated startle response compared to a context where the same shock was predictable (Fonteyne et al., 2010). Since the startle response is an ancestral defensive reflex, the amplitude of which is modulated by the emotional state of an individual (Lang, 1995), it can be considered as an implicit biopsychological measure of the individual's emotional state. Thus, threatening situations prime defensive responses and cause potentiation of startle amplitude, while appetitive situations cause startle amplitude attenuation (Fendt and Fanselow, 1999; Koch, 1999). Hence, these findings suggest that the simple unpredictability of an aversive event increases the experienced aversiveness as indicated by explicit and implicit measures.

The present study moved one step further to examine if and how the unpredictability of an aversive event affects the relief experienced after its offset. According to previous findings, pain relief is appetitive and organisms react with reward-like responses to stimuli associated with it. Namely, humans show reward-like brain activations (e.g., ventral striatum) to a stimulus temporally contiguous to the decrease (Seymour et al., 2005) or the omission (Leknes et al., 2011) of a painful stimulation. Moreover, conditioned responses to a relief-associated stimulus are similar to those to a reward-associated stimulus. That is, appetitive events or stimuli predicting these events induce attenuation of the startle response (Schneider and Spanagel, 2008), or activation of the ventral striatum (Gottfried et al., 2002). Comparably, fruit flies avoid an odor (conditioned stimulus, CS) which was repeatedly presented before a painful unconditioned stimulus (US; forward conditioning or fear conditioning) but approach an odor which repeatedly followed a painful US (backward conditioning or pain relief conditioning; Tanimoto et al., 2004; Yarali et al., 2008). In the same vein, rats show after the injury of the muscle of a paw conditioned place preference (CPP) for the chamber in which the pain was alleviated by a local anesthesia (Navratilova et al., 2012). Finally, rats and humans respond with startle attenuation to a stimulus associated with pain offset, and such relief-associated stimulus activate striatal regions (Andreatta et al., 2010, 2012). Therefore, the relief reaction which follows a painful stimulation seems to entail appetitive properties.

These results support the opponent-process theory of Solomon (1980) and the relaxation theory of Denny (1971) which assert that aversive or painful events are initially characterized by a negative emotional state determined by the aversiveness of the pain itself. However, as soon as such aversive stimulation terminates, individuals feel an emotional state which entails opponent, namely appetitive properties. In line, the pleasantness of pain relief is linearly correlated with the aversiveness of the preceding painful stimulation that is the more the pain intensity is increased the more positive the following relief is experienced (Leknes et al., 2008). Considering the etiology of anxiety disorders (Mineka and Zinbarg, 2006), avoidance behavior is frequently considered to be maintained by negative reinforcement due to the relief following such behaviors (Kim et al., 2006). Besides such operant conditioning, classical conditioning may play an important role too since stimuli associated with the relief very likely guide behaviors (i.e., safety behavior). In any case, it is of crucial importance to unravel the impact of relief on conditioned responses and behaviors because this may in the long run allow improving therapeutic intervention of anxiety disorders.

Despite the appetitive physiological and neural responses, humans may value a stimulus associated with relief as negatively valenced and high arousing. We found that the verbal and explicit ratings of the participants dissociated from the physiological/neural and implicit responses (Andreatta et al., 2010, 2012). This dissociation can be understood on the basis of psychological theories which posit two systems: an impulsive and a reflective system, which can work in a synergic or antagonist fashion (Strack and Deutsch, 2004). The impulsive system generates behaviors on the basis of automatic processes influenced by simple associative learning mechanisms, while the reflective system generates behaviors on the basis of explicit knowledge about the situation. It is then presumable that the physiological and neural responses to a relief-associated CS are mediated by the impulsive system, but the ratings by the reflective system, which seems to consider the temporal contiguity of the US more important than the ongoing appetitive reaction. Supportively, human participants in our previous studies reported the stimulus presented upon pain termination—that is at the moment of relief—as being temporally linked to the painful US (Andreatta et al., 2010, 2012).

Because pain aversiveness is modulated by its predictability and because appetitive properties of pain relief depend on pain aversiveness, we assume that the predictability of pain modulates the following relief as well. In line with our previous studies (Andreatta et al., 2010, 2012), we hypothesize that participants show attenuated startle amplitude (i.e., reward-like responses) to a relief-associated stimulus. We further hypothesize that startle amplitude would be more attenuated for stimuli associated with the offset of unpredictable vs. predictable painful USs. Moreover, we expect a dissociation between physiological and verbal responses to a stimulus associated with the offset of an US which is delivered unpredictably as in our previous between-subjects designed studies; that is negative valence and high arousal ratings (Andreatta et al., 2010, 2012). On the contrary, we expect positive ratings of the relief-associated CS when it follows a predictable US, because in this case participants would not explicitly associate the relief-associated CS with the painful US. In order to investigate these hypotheses, we conducted two studies in which we presented one stimulus as signal for pain onset (_FORWARD_CS+) and another stimulus upon the moment of the relief (i.e., after pain offset, _BACKWARD_CS+). In the first study, participant could predict only half of the painful USs, whereas the other half was delivered unwarned. In the second study, participants could reliably predict all painful USs. In both studies, we measured startle responses and skin conductance response (SCR) to conditioned visual stimuli as indices of implicit and physiological learning. In addition, we collected verbal reports for the valence and the arousal of the visual stimuli as indices of explicit and cognitive learning.

## Study 1

In Study 1 we investigated whether the unpredictability of a painful event (US) would induce reward-like physiological responses but negative reports. In other words, we wanted to replicate the results of our previous between-subjects study (Andreatta et al., 2010) in a within-subjects study. To this purpose, each participants experienced sixteen USs which were presented predictably at the offset of one visual stimulus (_FORWARD_CS+) and 16 USs which were delivered unpredictably shortly before another visual stimulus (during relief, _BACKWARD_CS+).

### Materials and methods

#### Participants

Forty-one volunteers participated in the study and were recruited through media advertisements. For their participations, individuals received 14€. Eleven participants were excluded from the analysis. Three participants were excluded because they lost the electrodes for the electric shock (US) during the experiment and one because of technical problems. Seven additional participants were excluded from the analysis, one because interrupted the experiment, three because they were coded as non-responders (mean startle amplitude <5 μV) and three because they did not have enough startle responses per condition (minimum = 4; for details see Materials and Methods). At the end, we considered 30 participants for the analysis (16 males; mean age: 25.33 years, *SD* = 3.18; range = 20–33 years).

#### Stimulus material

The aversive US consisted of a mild painful electric shock (200 ms duration). The shock was an electric pulse delivered with a frequency of 50 Hz. The intensity of the shock was individually assessed with a threshold procedure consisting of two ascending and descending series of electric shocks in steps of 0.5 mA (for details see Andreatta et al., 2010). The electric shock was generated by a current stimulator (Digitimer DS7A, Digitimer Ltd, Welwyn Garden City, UK, 400 V, maximum of 9.99 mA) and delivered by two disk electrodes with 9 mm diameter and spacing 30 mm over the forearm of the dominant hand. Participants rated the subjective painfulness of the US by means of a scale ranging from 0 (“feeling nothing at all”) to 10 (“very intense pain”) with 4 as an anchor for “just noticeable pain.” The mean value of painful intensities was then increased by 1 mA. The mean intensity of the US was 2.32 mA (*SD* = 0.64) while the subjective intensity was 6.30 (*SD* = 1.51).

As visual *conditioned stimuli* (CS) we used yellow geometrical shapes presented for 8 s on a 19″ computer screen localized circa 80 cm in front of the participants at the eye level over a black background. Shapes were a square, a triangle, a circle and a hexagon with 7.8 cm width and 7.8 cm height. The inter-stimulus interval (ISI), defined as the time between CS onset and US onset was as follows: the US was delivered either at the offset of one shape (_FORWARD_CS+; ISI = 8 s) or 6 s before the onset of another shape (_BACKWARD_CS+; ISI = −6 s). During the conditioning phase, three shapes were presented: _FORWARD_CS+, _BACKWARD_CS+ and a third shape (CS−), which was never associated with the US. During the test phase, four shapes were presented: _FORWARD_CS+, _BACKWARD_CS+, CS−, and a novel shape (NEW) as control stimulus. Shapes were counterbalanced across participants.

The *startle probe* was a burst of white noise of 98 dB with duration of 50 ms. The acoustic stimuli were presented binaurally over headphones and occurred randomly 3–7 s after shape's onset.

Two *questionnaires* were used as indicators for anxiety traits and the actual emotional state of the participants. The German version of the State-Trait Anxiety Inventory (STAI, Laux et al., 1981) is an inventory to assess the trait and/or the state anxiety of the participants. Both the trait and the state version consist of 20 items, respectively. Participants had to rate on a 4-point Likert scale from 1 (“almost never”) until 4 (“almost always”) how much the item would describe their anxiety. Higher scores indicate greater anxiety. Participants' anxiety level before and after the experiment did not change significantly [35.4 ± 4.8 vs. 35.7 ± 4.6; *t*_(29)_ = 0.31, *p* = 0.758]. Trait anxiety scores in the current sample ranged between 20 and 58 (mean = 36.7, *SD* = 8.64), which is comparable to the published normal range of adults (Laux et al., 1981). The Positive and Negative Affect Schedule (PANAS; Krohne et al., 1996) is a questionnaire to asses participants' mood. High scores on the PA scale reflect positive affectivity and individuals are disposed to emotions such as enthusiasm. While high scores on the NA scale represent negative affectivity and individuals are disposed to emotions such as distress. Participant had to indicate to what extend he/she feels a particular emotion on a scale ranging from 1 (“very slightly”) to 5 (“extremely”). Participants negative affect did not change throughout the experiment significantly [13.07 ± 5.4 vs. 11.60 ± 2.6; *t*_(29)_ = 1.56, *p* = 0.129], but they reported less positive mood at the end of the experiment in comparison to the beginning [28.9 ± 4.7 vs. 24.97 ± 5.3; *t*_(29)_ = 4.42, *p* < 0.001]. Such decrease of participant's positive mood might have depended on the unpleasantness of the paradigm (painful electric shock as well as an aversive white noise were presented).

#### Procedure

Upon the arrival in the laboratory, participants read and signed an informed consent approved by the ethics committee of the Deutsche Gesellschaft für Psychologie (DGPs), in which they were informed that a series of geometrical shapes, an electric shock and loud noises will have been presented and that they should keep the shapes in their visual focus. We did not mention the contingency between CSs and US. After having filled in the questionnaires, the electrodes were attached and the pain threshold procedure was performed as described above.

**Figure 1:**
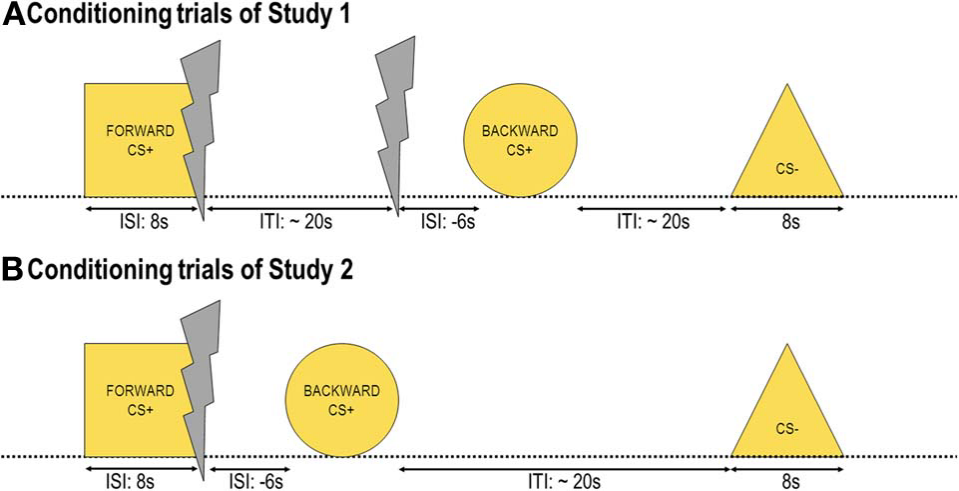
**Conditioning trials.** Three out of four yellow geometrical shapes were presented during conditioning as conditioned stimuli (CS). One shape (_FORWARD_CS+) was presented before a painful electric shock (unconditioned stimulus, US), one shape (_BACKWARD_CS+) was presented after the US, and another shape (CS−) was never associated with the US. In Study 1 **(A)** 16 USs out of 32 were predicted by the _FORWARD_CS+, whereas the other 16 USs were delivered unwarned before the _BACKWARD_CS+. In Study 2 **(B)** all USs were preceded by the _FORWARD_CS+ and followed by the _BACKWARD_CS+.

The experiment consisted of two phases: The conditioning and the test phase separated by subjective ratings. During the *conditioning phase* (Figure 1A) participants saw three out of four geometrical shapes 16 times each. Altogether, there were 48 trials, 16 CS− trials, 16 _FORWARD_CS+ trials, and 16 _BACKWARD_CS+ trials. The inter-trial interval (ITI) defined as the time between stimulus offset and the subsequent stimulus onset varied between 20 and 30 s (mean = 25 s). The choice of this relatively long ITI was made in accordance with our previous study (Andreatta et al., 2010) as well as to avoid carry-over effects from one trial to the following one. Stimulus presentation was randomized with the only restriction that the same stimulus may not be presented more than twice in a row. No startle probe was presented during conditioning.

Before the test phase, 7 white noises were delivered every 7–15 s in order to decrease the initial startle reactivity. During the *test phase* participants saw four geometrical shapes, that were the three CSs (_FORWARD_CS+, _BACKWARD_CS+, and CS−) and a novel neutral shape (NEW) as control stimulus. No US was delivered during the test phase. Each stimulus was presented 16 times in a pseudorandom order (i.e., the same stimulus was not presented more than twice consequently), so altogether there were 64 trials. During the test phase, for 8 of the 16 stimulus presentations a startle probe was delivered between 3 and 7.5 s after stimulus onset in order to provoke the automatic defensive reflex. As in the conditioning phase, the ITIs varied between 20 and 30 s. In order to assure the unpredictability of the startle probes we additionally delivered 8 startling noises during the ITIs.

Before and after the conditioning phase as well as after the test phase, participants had to rate the valence (pleasantness) and the arousal (excitatory) of the visual stimuli by using two different visual analog scales (VAS) ranging from 1 until 9. One indicates “very unpleasant” for the valence and “calm” for the arousal, while 9 indicates “very pleasant” and “exciting,” respectively. In addition, after the conditioning phase we verified participants' contingency awareness with a VAS ranging from 0 (no association) until 100 (perfect association). The contingency awareness indicates participant's ability to verbally report the association between the _FORWARD_CS+, _BACKWARD_CS+ or CS−, and the US.

#### Physiological recording and data reduction

Physiological responses were recorded with a V-Amp 16 amplifier and Vision Recorder V-Amp Edition Software (Version 1.03.0004, BrainProducts Inc., Munich, Germany). A sampling rate of 1000 Hz and a 50 Hz notch filter were applied. The offline analyses of these responses were conducted with Brain Vision Analyzer (Version 2.0; BrainProducts Inc., Munich, Germany).

Startle response was measured by means of electromyography (EMG) at the left *orbicularis oculi* muscle with two 5 mm Ag/AgCl electrodes. According to the guidelines (Blumenthal et al., 2005), one electrode was positioned under the pupil and the second one 1 cm laterally. The ground and the reference electrodes were placed on the right and left mastoids respectively. Before attaching the electrodes, the skin was slightly abraded and cleaned with alcohol in order to keep the impedance below 8 kΩ. EMG activity was continuously recorded. The electromygraphic signal was offline filtered with a 28 Hz low cutoff filter and a 500 Hz high cutoff filter as well as with a 50 Hz notch filter. Then the EMG signal was rectified and a moving average of 50 ms was applied. As baseline we used the 50 ms before startle probe onset (Grillon et al., 2006). Responses to startle probes were scored manually, and trial with excessive baseline shifts (±5 μV) or movement artifacts were excluded from further analysis. Altogether, 19.1% of the trials were rejected, and a minimum of 4 out of 8 startle responses for each condition was required to keep the participant for further analysis. The peak amplitude was defined as the maximum peak relative to baseline during the 20–120 ms time window after startle probe onset. The raw data were then normalized within-subjects using *z*-scores in order to reduce the influence of the individual variability and to better detect the psychological processes. The *z*-scores were averaged for each condition (_FORWARD_CS+, _BACKWARD_CS+, CS−, NEW, and ITI). In order to investigate startle potentiation or startle attenuation, the scores for the ITI startle responses were subtracted from the startle responses of each condition.

SCR was recorded using two 5 mm Ag/AgCl electrodes placed on the palm of the no-dominant hand. SCR was continuously recorded with the same V-Amp system, which delivered a constant current of 0.5 V. Sampling rate was 1000 Hz. The galvanic response was offline filtered with 1 Hz high cutoff filter. The SCR was defined as difference (in μS) between the response onset (1–3 s after shape onset) and the response peak (Tranel and Damasio, 1994; Delgado et al., 2011). Trials containing startle probes were not considered for the analysis of the SCR. Responses below 0.02 μS were coded as zero. For SCR analysis of the conditioning phase, two further participants were excluded and for the SCR analysis during the test phase we excluded 10 further participants because they had no detectable SCR (non-responses) in each condition. The skin raw conductance data were then square root transformed in order to normalize the distribution and the scores were averaged for each condition separately for the conditioning (_FORWARD_CS+, _BACKWARD_CS+, CS−) and the test phase (_FORWARD_CS+, _BACKWARD_CS+, CS−, and NEW).

#### Data analysis

All data were analyzed with SPSS for Windows (Version 20.0, SPSS Inc.). Startle amplitude, valence, arousal and contingency ratings were separately analyzed with multivariate analysis of variance (MANOVA). For all dependent variables MANOVAs had as within-subjects factor stimulus (_FORWARD_CS+, _BACKWARD_CS+, CS−, and NEW). The SCR was separately analyzed for the conditioning (_FORWARD_CS+, _BACKWARD_CS+, and CS−) and the test phase (_FORWARD_CS+, _BACKWARD_CS+, CS−, and NEW) having stimulus as within-subjects factor. In the analysis for the valence and the arousal ratings, the within-subjects factor phase was added (T1: before conditioning, T2: after conditioning, T3: after test phase), as well as for the contingency ratings (T1: after conditioning, T2: after test phase). The alpha (α) level was set at 0.05 for all analyses. The effect size is reported as partial η^2^.

### Results

#### Valence ratings (Figure 2A, left panel)

The valence of the four CSs was differentially affected by conditioning as confirmed by the significant Stimulus × Phase interaction [*F*_(6, 23)_ = 2.77, *p* = 0.035, η^2^_*p*_ = 0.42]. According to follow up *t*-tests, the valence of the four geometrical shapes did not differ before conditioning (all *p*s > 0.42) and were rated as neutral (i.e., 5; all *p*s > 0.40). After conditioning, the _FORWARD_CS+ and the _BACKWARD_CS+ had comparable valence [*t*_(28)_ = 0.56, *p* = 0.579] which was significantly more negative than the valence of both the CS− [_FORWARD_CS+: *t*_(28)_ = 3.48, *p* = 0.002; _BACKWARD_CS+: *t*_(28)_ = 2.73, *p* = 0.011] and the NEW [_FORWARD_CS+: *t*_(28)_ = 3.08, *p* = 0.005; _BACKWARD_CS+: *t*_(28)_ = 2.38, *p* = 0.024]. Valence ratings between the CS− and the NEW did not differ [*t*_(28)_ = 1.65, *p* = 0.110]. After the test phase, the _FORWARD_CS+ valence [*t*_(28)_ = 1.86, *p* = 0.073] and the _BACKWARD_CS+ valence [*t*_(28)_ = 2.00, *p* = 0.055] remained slightly more negative than the CS− valence although these comparisons just failed to reach the significance level. The valence ratings for the NEW did not differ from the other stimuli (all *p*s > 0.26) and the valence of the _FORWARD_CS+ did not differ from the _BACKWARD_CS+ [*t*_(28)_ = 0.44, *p* = 0.663]. In summary, both the _FORWARD_CS+ and the _BACKWARD_CS+ acquired negative explicit valence after conditioning.

#### Arousal ratings (Figure 2B, left panel)

The arousal of the four CSs was differentially modulated by conditioning as the significant Stimulus × Phase interaction indicates [*F*_(6, 23)_ = 2.51, *p* = 0.051, η^2^_*p*_ = 0.40]. Follow-up *t*-tests revealed equal arousal ratings among the geometrical shapes (all *ps* > 0.26) before conditioning. After conditioning, the _FORWARD_CS+ [*t*_(28)_ = 1.98, *p* = 0.058], the _BACKWARD_CS+ [*t*_(28)_ = 1.97, *p* = 0.059], but not the NEW [*t*_(28)_ = 1.90, *p* = 0.098] were slightly rated more arousing than the CS−, although these tests just failed to reach the significance level. Moreover, the _FORWARD_CS+, the _BACKWARD_CS+, and the NEW did not differ regarding arousal ratings (all *p*s > 0.44). After the test phase, the _FORWARD_CS+ [*t*_(28)_ = 2.30, *p* = 0.029] and the _BACKWARD_CS [*t*_(28)_ = 2.39, *p* = 0.024] were rated more arousing than the CS−, but not to the NEW [*t*_(28)_ = 0.94, *p* = 0.354]. Notably, arousal ratings of the _FORWARD_CS+ did not differ significantly from those of the _BACKWARD_CS+ [*t*_(28)_ = 0.27, *p* = 0.787] and the NEW [*t*_(28)_ = 1.55, *p* = 0.133] after the test phase, and the _BACKWARD_CS+ was rated with higher arousal compared to the NEW [*t*_(28)_ = 2.20, *p* = 0.036]. In summary, the _FORWARD_CS+ and the _BACKWARD_CS+ were rated as high arousing stimuli after conditioning, and such ratings lasted until the end of the experiment.

#### Startle response (Figure 3A)

Analysis of the startle response revealed no significant main effect of stimulus [*F*_(3, 27)_ = 0.23, *p* = 0.875, η^2^_*p*_ = 0.03] indicating no differential responses to the _FORWARD_CS+, the _BACKWARD_CS+, the CS−, and the NEW. We, however, compared *z*-scores of the startle amplitudes to the four visual stimuli with the mean (i.e., 0) and found that only the _FORWARD_CS+ induced a significant potentiation of the startle response [*t*_(29)_ = 2.10, *p* = 0.044].

#### SCR (Figure 4A)

Analysis of the SCR revealed that the conditioning differentially affected the SCR to the CSs as reflected in the significant main effect of stimulus [*F*_(2, 26)_ = 15.72, *p* < 0.001, η^2^_*p*_ = 0.547]. Follow up *t*-tests indicated that the _FORWARD_CS+ elicited higher SCRs compared to the CS− [*t*_(27)_ = 3.69, *p* = 0.001] and to the _BACKWARD_CS+ [*t*_(27)_ = 5.71, *p* < 0.001]. Moreover, SCRs to the _BACKWARD_CS+ were significantly lower than to the CS− [*t*_(27)_ = 4.21, *p* < 0.001]. Analysis of the SCR during the test phase indicated successful extinction learning as all stimuli elicited comparable SCRs [*F*_(3, 17)_ = 0.15, *p* = 0.928, η^2^_*p*_ = 0.026]. In summary, the _FORWARD_CS+ elicited enhanced fear responses (i.e., high SCR), whereas the _BACKWARD_CS+ seems to be less arousing as indicated by low SCR.

#### Contingency awareness (Table 1)

Participants were aware about the contingency between the CSs and the US as indicated by a significant main effect stimulus [*F*_(2, 27)_ = 24.10, *p* < 0.001, η^2^_*p*_ = 0.641]. Follow up *t*-tests indicated that participants reported significant higher contingency ratings for the _FORWARD_CS+ [*t*_(28)_ = 6.89, *p* < 0.001] and the _BACKWARD_CS+ [*t*_(28)_ = 5.20, *p* < 0.001] than for the CS−. Furthermore, contingency ratings for the _FORWARD_CS+ and the _BACKWARD_CS+ did not differ [*t*_(28)_ = 1.88, *p* = 0.071]. In summary, participants recognized the associations between the _FORWARD_CS+ and the _BACKWARD_CS+ and the US.

### Discussion

**Figure 2:**
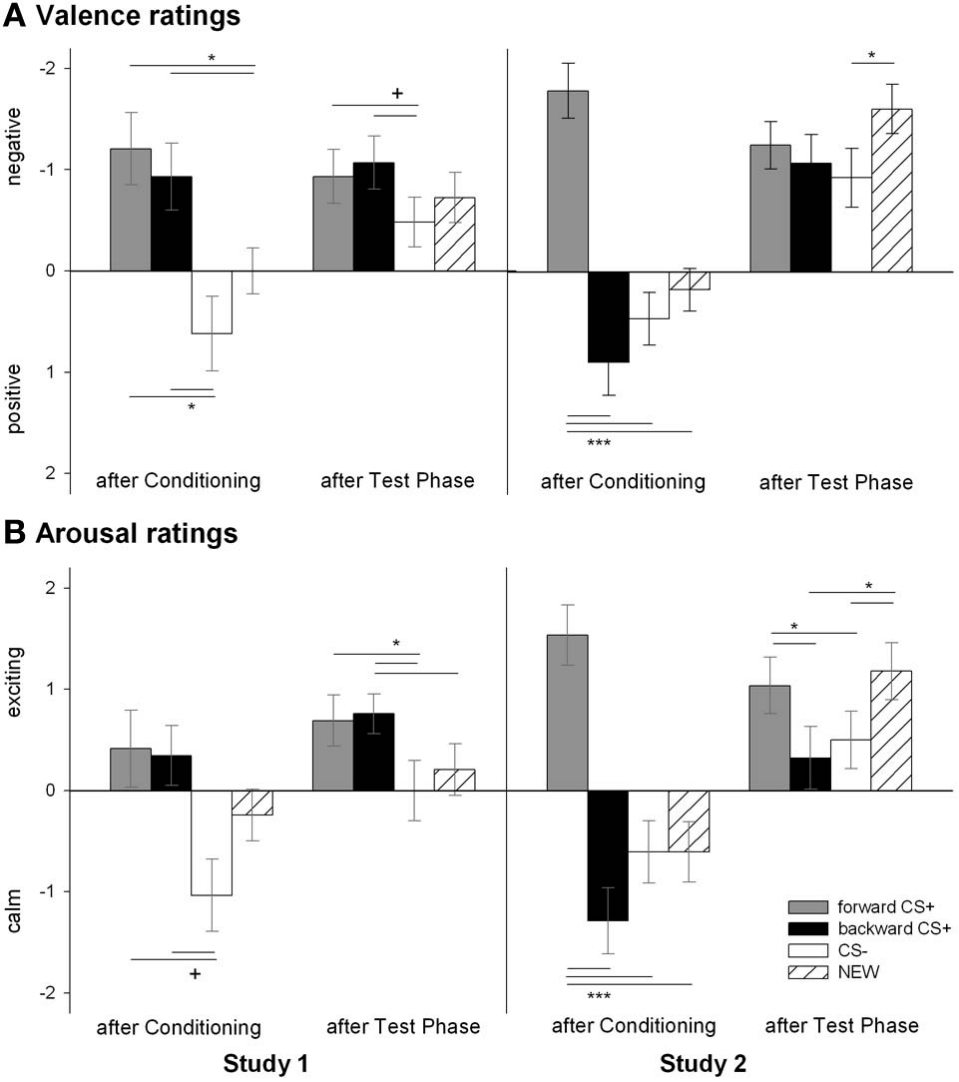
**Ratings of valence (A) and arousal (B) for the visual stimuli.** Participants rated the valence (upper panel) and the arousal (botton panel) of the _FORWARD_CS+ (light gray bars), the _BACKWARD_CS+ (black bars), the CS− (white bars), and the NEW (striped bars) before and after the conditioning as well as after the test phase. In Study 1 (left panels), both the _FORWARD_CS+ and the _BACKWARD_CS+ were rated as negatively valenced and arousing. On the contrary, in Study 2 (right panels) the _FORWARD_CS+ was rated as negatively valenced and high arousing, whereas the _BACKWARD_CS+ as positively valenced and low arousing (^+^*p* < 0.06; ^*^p < 0.05; ^***^p < 0.001).

The main goal of Study 1 was to investigate the modulatory role of the unpredictability of a painful electric shock (US) over relief. To this end, we presented half of the USs predictably after one visual stimulus (_FORWARD_CS+) while the other half was presented unwarned shortly before another visual stimulus (_BACKWARD_CS+). In line with our previous findings (Andreatta et al., 2010), we found that both the _FORWARD_CS+ and the _BACKWARD_CS+ compared to the CS− stimulus acquired explicit aversive properties through conditioning as indicated by negative valence and high arousal ratings (Figure 2). This acquired explicit aversiveness of both the _FORWARD_CS+ and the _BACKWARD_CS+ might be due to the cognitive knowledge that these two visual stimuli were temporally presented in association with the painful electric shock as the participants' contingency ratings indicate.

A further interesting and new result of this study is the modulation of the SCR by the CSs (Figure 4A). Namely, SCR to the _BACKWARD_CS+ was significantly lower compared to the _FORWARD_CS+ and the CS− during conditioning indicating that the relief-associated stimulus (_BACKWARD_CS+) was less arousing than the pain-signaling stimulus (_FORWARD_CS+) and even less arousing than the safety signal (CS−). These results are in line with previous human findings of low SCR during relief which also was positively correlated with the intensity ratings of the relief (Leknes et al., 2008). Notably, the physiological and the verbal responses dissociated. Namely, participants rated the _BACKWARD_CS+ as arousing, but they showed low SCR. Such dissociation is in line with the valence-related dissociation found in our previous studies (Andreatta et al., 2010, 2012). Thus, in the previous studies and the current study we found relief-like physiological responses (attenuation of startle amplitude and low SCR), but fear-like verbal reports (negative valence and high arousal). Apparently, the offset of unpredictable aversive stimuli is valued in an antagonist fashion by the implicit impulsive system and the explicit reflective system (Strack and Deutsch, 2004). On the one hand, the physiological responses reflect the implicit relief-reactions going on after a painful event. On the other hand, the explicit negative valuation may be imposed by the explicit knowledge that the stimulus is somehow associated with pain (see contingency rating, Dunsmoor et al., 2011). Finally, SCR did not differ among the _FORWARD_CS+, the _BACKWARD_CS+, the CS−, and the NEW during the test phase and this may be due to processes linked to extinction learning (Phelps and Ledoux, 2005).

**Figure 3:**
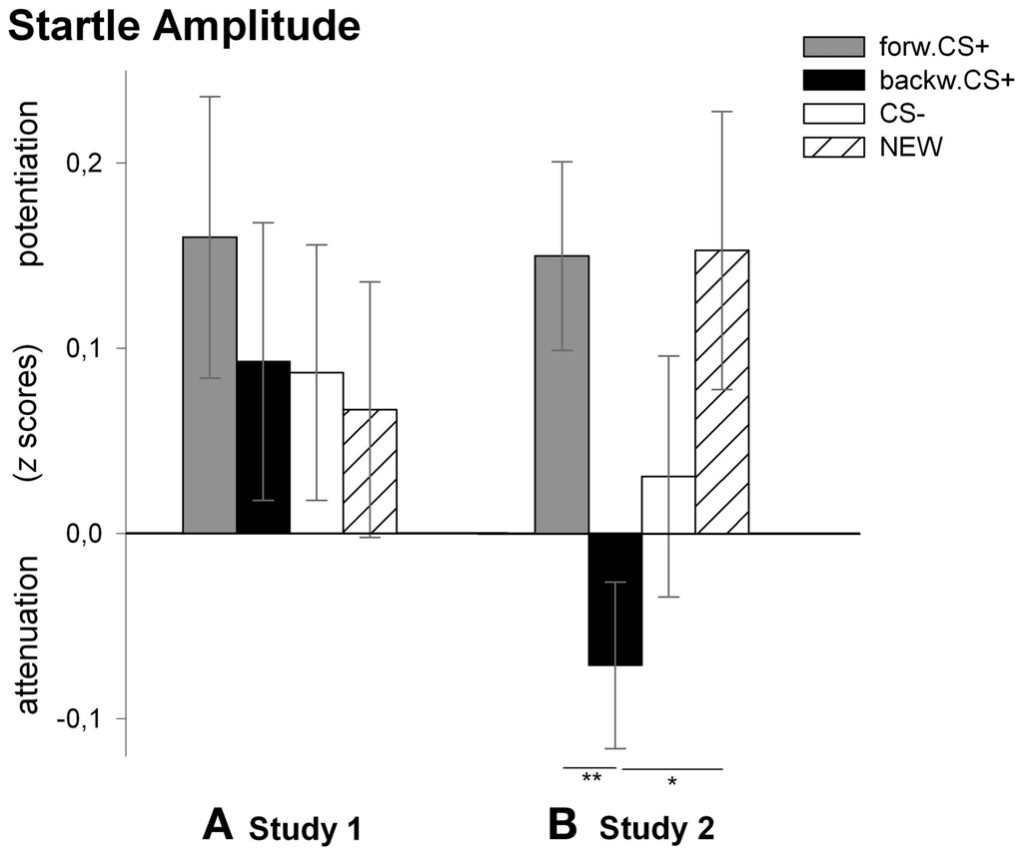
**Startle amplitudes to the visual stimuli during the test phase.** Bars (with standard errors) depict the startle amplitude in *z* scores in response to the _FORWARD_CS+ (light gray), the _BACKWARD_CS+ (black), the CS− (white), and the NEW (striped). Startle responses did not differ among CSs after conditioning in Study 1 **(A)**. On the contrary, startle response was significantly attenuated (i.e., reward-like conditioned responses) by the _BACKWARD_CS+ as compared to the _FORWARD_CS+ and the NEW after conditioning in Study 2 **(B)** (^*^p < 0.05; ^**^p < 0.01).

In contrast to our previous between-subjects designed study (Andreatta et al., 2010), we did not find any difference in the participants' startle responses to the CSs (Figure 3A). Most likely, differences in the number of the painful electric shocks together with their unpredictability might have played a crucial role here. In fact, we doubled the number of shocks in the present study compared to the previous between-designed studies (32 vs. 16). According to Fanselow and Lester (1988), circa-strike defensive responses depend on the shock density as well as on their imminence. Shock density refers to the number of shocks per time; the more dense the shock schedule is (i.e., the increased number of shocks), the more the animals present circa-strike defensive response (i.e., flight/fight). The imminence refers to the real presence and the vicinity of a danger, the closer a danger is the stronger fear responses are prompted. Referring to the present study, the US preceding the _BACKWARD_CS+ had no warning signal, which might have provoked a feeling of sustained fear or anxiety (Barlow, 2000; Grillon et al., 2008; Davis et al., 2010; Fonteyne et al., 2010). Moreover, both the USs presented after the _FORWARD_CS+ and those presented before the _BACKWARD_CS+ may have been experienced as one single aversive event, which was sometimes predicted, but sometimes not. Therefore, the unreliable prediction of the shock might have provoked a state of uncertainty which consequently induced anxiety rather than fear. Furthermore, the anxious feeling of the individuals in this study might have been stronger than the one induced in the between-designed study because of the higher density of the shocks. Hence, we think that the conditioned responses here are induced by a post-encounter stage rather than by a circa-strike stage, in line with Fanselow (1994) and Davis et al. (2010) who assume that post-encounter behavior resembles sustained anxiety, whereas circa-strike behavior is induced by phasic fear. Thus, participants have “encountered” the threat (the US), but because of its relative predictability, such threat is not sufficiently imminent for provoking clear discriminative fear responses (e.g., potentiation of the startle response to the threat signal).

## Study 2

In Study 2 we investigated the modulatory influence of a predictable pain over pain relief. For this purpose, we associated the painful electric shock (US) during all trials with both a _FORWARD_CS+ and a _BACKWARD_CS+. The _FORWARD_CS+ predicted all USs which were presented at its offset, and the _BACKWARD_CS+ followed all USs. Thus, here we never delivered an unpredictable painful US before the _BACKWARD_CS+, all USs were predictable by the _FORWARD_CS+. We expected appetitive conditioned responses to the _BACKWARD_CS+ as compared to the _FORWARD_CS+ such as attenuation of startle response and positive valence ratings. As opposed to Study 1, we did not expect a dissociation between implicit and explicit responses because in this case the _FORWARD_CS+ signals the US and consequently the _BACKWARD_CS+ might be explicitly associated with the its termination.

### Materials and methods

#### Participants

Thirty-three volunteers participated in the study and were recruited through media advertisements. For their participations, individuals received 14€. Three participants were excluded from the analysis: One because of technical problems, one because it interrupted the recording and the third one because it was the only one who was unaware (i.e., she was not able to indicate the association between the stimuli). Two additional participants were excluded from the analysis, because they were coded as no-responders (mean startle amplitude <5 μV). At the end, we considered 28 participants for the analysis (9 males; mean age: 22.96 years, *SD* = 1.48; range = 21–26 year). Participants' trait anxiety scores ranged between 24 and 65 (mean = 40.6, *SD* = 8.86), which is comparable to the published normal range of adults (Laux et al., 1981). Participants' anxiety level (STAI state) before and after the experiment did not change significantly [37.46 ± 6.77 vs. 39.75 ± 7.86; *t*_(27)_ = 1.31, *p* = 0.202] as well as negative affect [NA scale from PANAS; 12.50 ± 2.58 vs. 13.64 ± 4.99; *t*_(27)_ = 1.25, *p* = 0.223]. Similar to the Study 1, participants positive mood (PA scale from PANAS) significantly decreased at the end of the experiment compared to their mood at the beginning [28.18 ± 6.04 vs. 25.61 ± 7.23; *t*_(27)_ = 2.14, *p* = 0.041]. Again, this decreased positive mood may have been induced by the aversiveness of the stimuli used or by the boringness of the experiment.

#### Stimulus material

The stimulus material was exactly the same as in Study 1. The mean electric shock intensity was 1.84 mA (*SD* = 0.27) and participants' subjective painfulness of the US was 6.39 (*SD* = 1.26; range: 5–9). Importantly, participants still rated the US as painful at the end of the experiment (6.39, *SD* = 1.57; range: 3–10) and the two ratings did not differ [*t*_(26)_ = 0.33, *p* = 0.746].

#### Procedure

The procedure of the Study 2 was almost the same as in Study 1; the only difference was the number of USs and their predictability.

During the *conditioning phase* participants saw three out of four geometrical shapes 16 times each. Altogether, there were 32 trials, 16 CS− trials and 16 CS+ trials. The CS+ trials started with the _FORWARD_CS+ onset, at _FORWARD_CS+ offset the US was delivered (ISI = 8 s), and 6 s later the _BACKWARD_CS+ was presented (ISI = −6 s; Figure 1B). The CS− trials consisted of CS− presentation. The ITI varied between 20 and 30 s (mean = 25 s) for the same reasons as in Study 1 (see Page 6). Stimulus presentation was randomized with the only restriction that the same stimulus may not be presented more than twice in a row. No startle probe was presented during conditioning. The *test phase* and the *subjective rating* were exactly the same as in Study 1.

In addition, after conditioning we verified participants' awareness about the association between the CSs and the US by means of an open question. That is, participants had to verbally report to which geometrical shape the electric shock was associated. Only one participant recalled the association between the _BACKWARD_CS+ and the US, one participant was not able to indicate a particular shape (she was then coded as unaware and excluded from the statistical analysis), whereas all other participants recalled the association between the _FORWARD_CS+ and the US.

#### Physiological recording and data reduction

Physiological responses and data reduction worked out in exactly the same way as in Study 1. Notably, 9.2% of the trials were rejected for the analysis of startle response. Moreover, seven further participants were excluded from the analysis for the SCR during conditioning because they had no detectable peaks per condition and 12 for the same analysis during test phase.

#### Data analysis

All data were analyzed with SPSS for Windows (Version 20.0, SPSS Inc.) and as for the Study 1 startle amplitude, SCR, valence, arousal and contingency ratings were separately analyzed with MANOVA. Again the alpha (α) level was set at 0.05 for all analyses.

### Results

#### Valence ratings (Figure 2A, right panel)

Analysis of the valence ratings revealed a significant Stimulus × Phase interaction [*F*_(6, 22)_ = 5.32, *p* = 0.002, η^2^_*p*_ = 0.592]. *Post-hoc t*-tests indicated that the valence of the geometrical shapes at the beginning of the experiment was equally rated (all *ps* > 0.24) and that the valence was reported as neutral (i.e., 5; all *p*s > 0.28). After conditioning, the _FORWARD_CS+ was rated as more negatively valenced compared to the CS− [*t*_(27)_ = 5.97, *p* < 0.001], the NEW [*t*_(27)_ = 5.22, *p* < 0.001] and interestingly to the _BACKWARD_CS+ as well [*t*_(27)_ = 5.82, *p* < 0.001]. The valence ratings of the CS− did not differ significantly from those of the _BACKWARD_CS+ [*t*_(27)_ = 1.20, *p* = 0.242] and the NEW [*t*_(27)_ = 1.22, *p* = 0.234], and the _BACKWARD_CS+ was rated more positive than the NEW [*t*_(27)_ = 2.15, *p* = 0.041]. After the test phase, the valence ratings of the NEW were significant more negative than those of the CS− [*t*_(27)_ = 2.64, *p* = 0.014], but no other significant differences were found (all *p*s > 0.09). Contrarily to the Study 1, the relief-associated stimulus (the _BACKWARD_CS+) acquired explicit positive valence as opposed to the threat signal (the _FORWARD_CS+) and similar to the safety signal (the CS−).

#### Arousal ratings (Figure 2B, right panel)

Analysis of the arousal ratings revealed a significant modulation of conditioning as indicated by a significant Stimulus × Phase interaction [*F*_(6, 22)_ = 6.32 *p* = 0.001, η^2^_*p*_ = 0.633]. *Post-hoc t*-tests indicated equal arousal for all four CSs at the beginning of the study (all *p*s > 0.12). After conditioning, the _FORWARD_CS+ was rated as more arousing compared to the CS− [*t*_(27)_ = 5.30, *p* < 0.001], the NEW [*t*_(27)_ = 5.14, *p* < 0.001] and to the _BACKWARD_CS+ [*t*_(27)_ = 6.29, *p* < 0.001], while the _BACKWARD_CS+, the CS− and the NEW were rated with comparable arousal (all *p*s > 0.08). After the test phase, the _FORWARD_CS+ was still rated as more arousing than the CS− [*t*_(27)_ = 1.99, *p* = 0.057; despite marginally] and the _BACKWARD_CS+ [*t*_(27)_ = 2.50, *p* = 0.019], but not more arousing than the NEW [*t*_(27)_ = 0.72, *p* = 0.475] anymore. Moreover, the NEW was rated as significantly more arousing compared to the _BACKWARD_CS+ [*t*_(27)_ = 3.22, *p* = 0.003] and the CS− [*t*_(27)_ = 2.81, *p* = 0.009]. Notably, the CS− and the _BACKWARD_CS+ did not differ regarding arousal ratings [*t*_(27)_ = 0.50, *p* = 0.624]. Contrarily to the Study 1, the relief-associated stimulus (_BACKWARD_CS+) was valued as less arousing than the threat stimulus (_FORWARD_CS+).

#### Startle response (Figure 3B)

Analysis for the startle responses revealed a significant main effect of stimulus [*F*_(3, 25)_ = 3.85, *p* = 0.022, η^2^_*p*_ = 0.316]. *Post-hoc t*-tests indicated that the startle amplitude to the _BACKWARD_CS+ was significantly attenuated compared to the _FORWARD_CS+ [*t*_(27)_ = 2.85, *p* = 0.008] and to the NEW [*t*_(27)_ = 2.45, *p* = 0.021], but not to the CS− [*t*_(27)_ = 1.13, *p* = 0.267]. Moreover, the startle responses to the CS− did not differ significantly from those to the _FORWARD_CS+ [*t*_(27)_ = 1.53, *p* = 0.137] and to the NEW [*t*_(27)_ = 1.00, *p* = 0.326]. Because we did not find significant discriminative responses to the _FORWARD_CS+ and the CS−, we compared the *z*-scores of the startle amplitudes to the four visual stimuli with the mean (i.e., 0) in order to verify whether startle amplitude to the _FORWARD_CS+ was potentiated. Tests revealed significant startle potentiation to the _FORWARD_CS+ [*t*_(27)_ = 2.93, *p* = 0.007] and to the NEW [*t*_(27)_ = 1.59, *p* = 0.052; despite marginally], but not to the _BACKWARD_CS+ [*t*_(27)_ = 1.59, *p* = 0.123] and to the CS− [*t*_(27)_ = 0.47, *p* = 0.640].

#### SCR (Figure 4B)

Analysis for the SCR during conditioning revealed a significant main effect of stimulus [*F*_(2, 19)_ = 12.05, *p* < 0.001, η^2^_*p*_ = 0.559]. Similar to the Study 1, *post-hoc t*-tests indicated significant higher SCR to the _FORWARD_CS+ compared to the CS− [*t*_(20)_ = 3.15, *p* = 0.005] and to the _BACKWARD_CS+ [*t*_(20)_ = 4.91, *p* < 0.001]. Again, the SCR to the _BACKWARD_CS+ was lower compared to the CS− [*t*_(20)_ = 3.12, *p* = 0.005]. Same as for Study 1, analysis of the SCR during the test phase did not reveal a significant main effect of stimulus [*F*_(3, 13)_ = 2.24, *p* = 0.132, η^2^_*p*_ = 0.34].

#### Contingency awareness (Table 1)

Participants' awareness about the association between the visual stimuli and the painful shock was significantly modulated by conditioning as the significant main effect of stimulus indicated [*F*_(2, 19)_ = 92.87, *p* < 0.001, η^2^_*p*_ = 0.907]. *Post-hoc t*-tests indicated higher contingency ratings for the _FORWARD_CS+ compared to the _BACKWARD_CS+ [*t*_(20)_ = 10.99, *p* < 0.001] and to the CS− [*t*_(20)_ = 13.86, *p* < 0.001]. No significant difference between the _BACKWARD_CS+ and the CS− [*t*_(20)_ = 0.06, *p* = 0.951] was found.

### Discussion

In Study 2 we investigated whether a stimulus associated with the relief from a painful US would acquire reward-like properties even when the aversive event is fully predicted. This is exactly what we found. Thus, when the _BACKWARD_CS+ followed a fully predictable painful US, participants showed significant attenuation of the startle amplitude (i.e., reward-like responses) to the _BACKWARD_CS+ compared to the _FORWARD_CS+ and the NEW stimulus (Figure 3B). Therefore, when the onset of the US was predictable, the _BACKWARD_CS+ appears to acquire implicit positive valence in parallel to our previous findings (Andreatta et al., 2010). Strikingly and in contrast to our previous studies (Andreatta et al., 2010, 2012), the _BACKWARD_CS+ in this case acquired an explicit positive valence too and low arousal (see Figure 2).

Why does the _BACKWARD_CS+ acquire explicit appetitive properties when presented after a _FORWARD_CS+, but explicit aversive properties when presented “alone”? Differently from Study 1, participants might have felt in Study 2 less anxious since the threat was fully predictable. Moreover, all participants (except one) explicitly indicated the _FORWARD_CS+ and not the _BACKWARD_CS+ as the visual stimulus associated with the US (see contingency ratings, Table 1). The absence of an explicit association between the _BACKWARD_CS+ and the US may have determined its positive valence and its low arousal ratings. Thus, since the _FORWARD_CS+ reliably predicted the US, the _BACKWARD_CS+ became explicitly associated with the relief only.

**Table 1:** **Contingency ratings**.

	**After conditioning**		**After test phase**	
	**Study 1**	**Study 2**	**Study 1**	**Study 2**
_FORWARD_CS+	83.45 (24.39)	97.14 (11.02)	70.69 (36.83)	84.76 (33.56)
_BACKWARD_CS+	71.72 (31.97)	7.62 (19.98)	65.17 (37.57)	8.57 (23.93)
CS−	20.00 (27.52)	8.10 (18.61)	24.14 (32.35)	8.57 (18.24)

Scores (standard deviation) indicate the subjective expectancy of the painful US in association with the respective shapes. Zero indicated “no association at all” and 100 “perfect association.”

We did not find during the test phase discriminative startle responses to the _FORWARD_CS+ and the CS−. This result is quite puzzling considering the broad literature on classical fear conditioning. However, we should consider that the startle responses were recorded during the test phase in which no USs were delivered. Therefore, it is possible to assume that a new learning (i.e., extinction learning) has started and modulated these responses (Milad and Quirk, 2012). In any case, the strong attenuation of the startle response to the _BACKWARD_CS+ suggests that relief-conditioned responses undergo slower extinction processes; although, this hypothesis must be further investigated.

**Figure 4:**
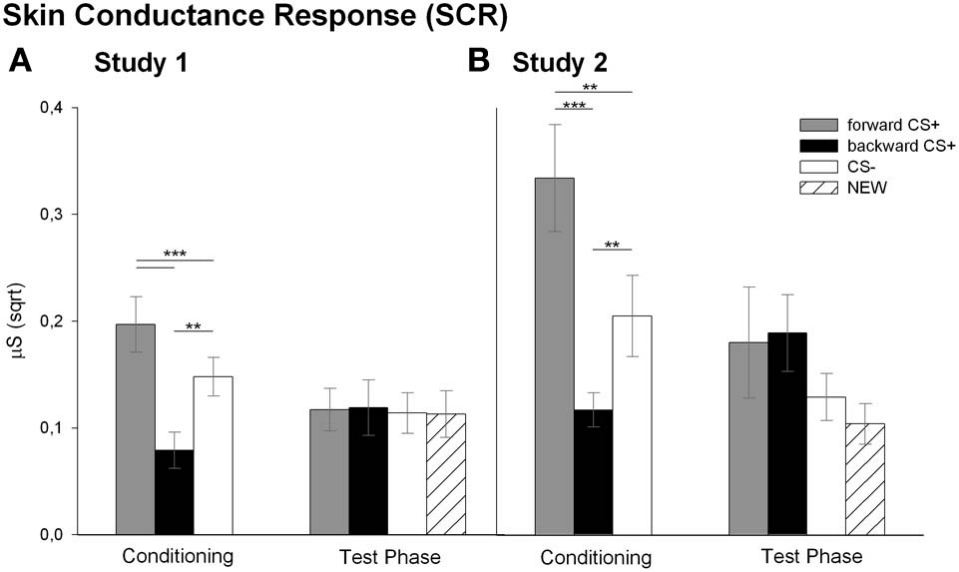
**Skin conductance responses (SCRs) to the visual stimuli.** Bars (with standard errors) depict the SCR (*sqrt* transformed) in response to the _FORWARD_CS+ (light gray), the _BACKWARD_CS+ (black), the CS− (white) and the NEW (striped) either during the conditioning or during the test phase. In both Study 1 **(A)** and Study 2 **(B)** the SCR to the _BACKWARD_CS+ was significantly lower compared to the _FORWARD_CS+ and the CS− (^**^p < 0.01; ^***^p < 0.001).

Nicely, the SCR findings in Study 2 mirror the SCR results of Study 1. Namely, SCR to the _BACKWARD_CS+ was significantly lower compared to the other CSs during conditioning (Figure 4B). Moreover, the greater SCR to the _FORWARD_CS+ compared to the CS− confirms previous studies, in which participants showed increased SCR to the threat-predicting CS suggesting greater sympathetic arousal (Büchel et al., 1998; Labar et al., 1998; Weike et al., 2008; Li et al., 2011). Finally, it is conceivable that pain relief does not implicate or does not need strong sympathetic engagement because there is no real need to react since the threat is not imminent anymore (see General Discussion for further interpretations; Fanselow and Lester, 1988; Fanselow, 1994). Discriminative SCRs to the CSs disappeared during the test phase, which may be related to extinction processes.

## General discussion

The goal of the present studies was to investigate the temporal sequence between pain and its relief and how their contiguity and predictability would affect the individuals' implicit and explicit responses. Because of the dependence of relief pleasantness on pain aversiveness and of pain aversiveness on pain unpredictability, we wondered whether the prediction of a painful stimulus might differentially modulate the responses to a stimulus associated with pain relief. We realized two studies which were similar in most aspects, but differed in the predictability of the painful US. During the conditioning phase of both studies, one geometrical shape (_FORWARD_CS+) was presented *before* a mild painful electric shock (aversive US), while another geometrical shape (_BACKWARD_CS+) was presented *after* the US, and a third geometrical shape (CS−) was unrelated to the US. In Study 1 on the one hand, the _FORWARD_CS+ and the _BACKWARD_CS+ were presented in different trials meaning that during the _FORWARD_CS+ trials the US could be predicted, while during the _BACKWARD_CS+ trials the _BACKWARD_CS+ followed an unpredicted US. In Study 2 on the other hand, the _FORWARD_CS+ and the _BACKWARD_CS+ were presented in one trial meaning that the US could be predicted by the _FORWARD_CS+ and the _BACKWARD_CS+ followed this predictable US.

Based on our previous and other fear conditioning studies, we hypothesized that the _FORWARD_CS+ would acquire negative affective implicit and explicit properties in both studies. This assumption was confirmed. Participants showed increased fear responses to the _FORWARD_CS+ as indicated by potentiation of the startle response, high SCR, and negative valence as well as enhanced arousal ratings^1^. In other words, the _FORWARD_CS+ acquired aversive explicit and implicit properties by means of its association with the painful US; it became a signal of danger (Weike et al., 2008; Andreatta et al., 2010; Delgado et al., 2011; Li et al., 2011).

For Study 1, we expected in line with our previous between-subjects studies that the _BACKWARD_CS+ would acquire an implicit positive valence because of its coincidence with the experience of relief, but an explicit negative valence because it is the only stimulus which was contiguous to the US. These hypotheses were confirmed for rating data, but not for startle data (for a discussion of the discrepancy in startle data between Study 1 and previous studies see Discussion of Study 1). In contrast and most important, we expected for Study 2 that the _BACKWARD_CS+ would acquire an implicit positive valence because of its coincidence with the experience of relief, and we also expected explicit positive properties because this stimulus should be experienced as independent from the US on a cognitive level. These hypotheses were confirmed. Thus, startle response was attenuated in response to the _BACKWARD_CS+ and participants reported positive valence as well as low arousal ratings for the _BACKWARD_CS+.

Learning the relationship between a neutral stimulus (CS) and an aversive event (US) implicates two kinds of memories (Williams et al., 2001; Hamm and Weike, 2005; Riebe et al., 2012). On the one hand, organisms form implicit fear memories which activate subcortical structures of the fear matrix like the amygdala and initiate defensive responses in an automatic manner, i.e., without cognitive appraisal. On the other hand, organisms form explicit fear memories which involve cortical structures like prefrontal cortex (PFC) and initiate fear responses requiring cognitive appraisal. Intuitively, the explicit cognitive knowledge about CS−US association may strongly influence participants' verbal reports, but to a lesser extend implicit memories. Hence, it is plausible that a stimulus presented upon the moment of the relief may acquire either aversive or appetitive explicit properties dependent on declarative encoding of the CS−US relation. In line, in Study 1 as well as in our previous studies (Andreatta et al., 2010, 2012) participants received an unpredictable aversive stimulus which was shortly followed by the _BACKWARD_CS+. We think that after an aversive event an appetitive reaction is always started, but the explicit encoding of such appetitive reaction is determined by the declarative processing of the temporal relationship between the stimuli. Hence, the impossibility to reliably foresee the aversive event entailed a negative valuation of all stimuli which were temporally nearby the event. On the contrary in Study 2, participants were able to reliably predict the aversive event by a preceding stimulus. Consequently, participants may have experienced the _BACKWARD_CS+ as “purely” associated with the relief because there was no need for an association between the painful event and any following stimuli. This interpretation is supported by the contingency ratings. In fact, if the US was unpredictable as in Study 1 and in our previous studies (Andreatta et al., 2010, 2012), participants reported an association between the US and the _BACKWARD_CS+. If the US was predicted by a preceding CS as in Study 2, participants report no contingency between the US and the _BACKWARD_CS+. As results, the synergic information from the implicit and the explicit level allows the participants to rate the _BACKWARD_CS+ as appetitive (i.e., positive valence) and reassuring (i.e., low arousal).

As already mentioned, pain relief entails reward-like properties (Seymour et al., 2005; Leknes et al., 2008, 2011) and promotes appetitive learning (Tanimoto et al., 2004; Yarali et al., 2008; Andreatta et al., 2010, 2012; Navratilova et al., 2012). That is, brain areas involved in the processing of rewarding events (Tobler et al., 2003) are also activated by pain relief (Seymour et al., 2005; Leknes et al., 2011), and organisms react with appetitive conditioned responses to a stimulus presented upon the relief (Tanimoto et al., 2004; Yarali et al., 2008; Andreatta et al., 2010, 2012; Navratilova et al., 2012). Confirming these studies, we found discriminative conditioned responses to the CSs in Study 2 (i.e., potentiation of the startle response to the _FORWARD_CS+ and attenuation of the startle response to the _BACKWARD_CS+), but not in Study 1. Why? First, the number of the shocks in Study 1 was doubled compared to Study 2 (32 vs. 16) and to our previous studies with between designs (Andreatta et al., 2010, 2012). Second, in Study 1 half of the shocks were reliably predictable, whereas the other half was delivered unpredictably before the _BACKWARD_CS+, while in Study 2 all USs were fully predictable. In the laboratory, fear responses can be induced by increasing the frequency of the shocks (shock density) together with their imminence (i.e., how reliably the danger is foreseen; Fanselow and Lester, 1988). Presumably, the high number of shocks in Study 1 together with their relative unpredictability might have induced an enhanced state of sustained fear (Davis et al., 2010) which caused the lack of discriminative startle responses to the CSs. In line, unpredictability, defined as “*the absence of a signal for an aversive event*” (Fonteyne et al., 2010), induces stronger fear responses to the aversive event than when it is predictable (Carlsson et al., 2006; Baratta et al., 2007; Herry et al., 2007; Fonteyne et al., 2010), a reduced capacity to identify safety periods (Lohr et al., 2007) and a sustained state of apprehension (sustained fear or anxiety; Fanselow and Lester, 1988; Davis et al., 2010). Moreover,the shock density seems also to play a role. In fact, we found relief-conditioned responses to a stimulus in the between-subjects designed studies (Andreatta et al., 2010, 2012), but not in Study 1 despite in both studies the US was presented unpredictably. Possibly, doubling the number of the aversive US may also have increased the anxiety-related responses. Hence, the unpredictability together with the high frequency of an aversive event seems to impair the ability to distinguish between threatening and safety periods.

Notably, these findings broaden our knowledge about the role of (un-)predictability of an aversive event in determining fear vs. safety conditioned responses. In fact, we could demonstrate for the first time that the unpredictability of an aversive event not only implies a sustained feeling of fear and an incapacity to identify the absence of a threat (i.e., respite), but it may also erase the ability to identify the termination of the threat (i.e., relief). That is, the stronger a sustained feeling of fear (or anxiety) is, the less evident the appetitive feeling of relief becomes. Safety is functionally related to danger meaning that an individual can identify safety periods only if it has first located the danger (Lohr et al., 2007). Based on this safety/danger relation, Lohr et al. (2007) distinguished between two kinds of safety, namely the absence of threat (i.e., respite) and the termination of threat (i.e., relief). Interestingly, a fruitless search for safety has been implicated in the etiology of anxiety disorders (Seligman, 1968; Mineka and Zinbarg, 2006; Lohr et al., 2007; Grillon et al., 2008, 2009; Davis et al., 2010), and anxious individuals have been found to be particularly sensitive to unpredictable threats (Grillon et al., 2008, 2009; Davis et al., 2010; Glotzbach-Schoon et al., 2013). Hence, individuals who show exaggerated fear responses to threatening contexts (Grillon et al., 2008, 2009; Davis et al., 2010) are less able to identify safety periods (Seligman, 1968; Mineka and Zinbarg, 2006; Lohr et al., 2007; Grillon et al., 2008, 2009; Davis et al., 2010), but they might also be less able to experience relief—as suggested by the present studies. However, further studies have to investigate the role of trait anxiety in the modulation of the relief-related responses in order to clarify whether and how the relief after an aversive event is implicated in the etiology of anxiety disorders.

Besides startle response, SCR is frequently used as physiological measure of conditioned fear (Büchel et al., 1998; Labar et al., 1998; Knight et al., 2003, 2006; Delgado et al., 2006; Weike et al., 2008). SCR reflects a phasic change in sweat gland activity induced by a re-orientation of the attentional resources toward novel and salient stimuli (Williams et al., 2000; Bradley et al., 2001; Bradley, 2009). In line with previous studies, both studies presented here found increased autonomic arousal to the danger signal (_FORWARD_CS+) compared to the safety signal (the CS−; Büchel et al., 1998; Labar et al., 1998; Knight et al., 2003, 2005, 2006; Weike et al., 2008; Alvarez et al., 2011), and these fear conditioned SCRs extinguished throughout the test phase. In parallel, the SCRs to the stimulus associated with the relief “signal” (_BACKWARD_CS+) were significantly reduced compared to the danger signal and even significantly reduced compared to the safety signal (CS−). The latter difference suggests that the processes triggered by a relief-associated stimulus differ from those underlying the processes of a stimulus signaling safety. To our knowledge, there is no evidence in the literature investigating the effects of conditioned pain relief on autonomic arousal, which makes the interpretation of our results quite difficult. Nevertheless, Leknes et al. (2008) showed that the electrodermal responses following a painful stimulus linearly decreased by the increase of painfulness. Furthermore, another possible explanation of the decreased SCR to the relief-associated stimulus might be linked to the imminence of the threat. Namely, the defensive pattern is determined by three stages defined on the imminence of threat (Fanselow, 1994). Thus, flight/fight responses are initiated by the physical contact with the threat, while the level of fear gradually decreases by danger detachment. Considering SCR as an index of physiological arousal, the low SCR in response to the _BACKWARD_CS+ during conditioning presumably relies on the evident termination or detachment of the painful stimulation (i.e., the US) and the no-need to initiate defensive responses.

In conclusion, our results concur with the growing evidences on the appetitive properties of pain relief and its conditionability. Importantly, the predictability and the cognitive appraisal of the association between two stimuli crucially affect the explicit aversiveness and pleasantness of the relief-associated stimulus. Thus, as soon as the danger (US) is reliably predicted by a stimulus (_FORWARD_CS+), another stimulus presented upon the termination of danger (_BACKWARD_CS+) can acquire not only implicit but also explicit appetitive properties linked to the experienced relief.

### Conflict of interest statement

The authors declare that the research was conducted in the absence of any commercial or financial relationships that could be construed as a potential conflict of interest.

## Footnotes

^1^Explorative we compared the subjective ratings, the startle response, and the SCR to the forward CS+ after conditioning of Study 1 vs. Study 2. We found no significant differences for SCR, startle response, valence and contingency ratings (*p*s > 0.07). However, forward CS+ in Study 2 was significantly more arousing compared to forward CS+ in Study 1 [*t*_(55)_ = 3.001, *p* = 0.004]. Supposedly, when a stimulus' reliability regarding a threat is partial, the arousal is spread over the other stimuli too as possible informers about the threat.
